# Determining the *N*-Representability of a Reduced Density Matrix via Unitary Evolution and Stochastic Sampling

**DOI:** 10.1021/acs.jctc.4c01166

**Published:** 2024-11-14

**Authors:** Gustavo E. Massaccesi, Ofelia B. Oña, Pablo Capuzzi, Juan I. Melo, Luis Lain, Alicia Torre, Juan E. Peralta, Diego R. Alcoba, Gustavo E. Scuseria

**Affiliations:** † Departamento de Ciencias Exactas, Ciclo Básico Común, Universidad de Buenos Aires, Ciudad Universitaria, 1428 Buenos Aires, Argentina; ‡ Instituto de Investigaciones Matemáticas “Luis A. Santaló” (IMAS), Consejo Nacional de Investigaciones Científicas y Técnicas, Universidad de Buenos Aires, Ciudad Universitaria, 1428 Buenos Aires, Argentina; § Instituto de Investigaciones Fisicoquímicas Teóricas y Aplicadas, Universidad Nacional de La Plata, Consejo Nacional de Investigaciones Científicas y Técnicas, Diag. 113 y 64 (S/N), Sucursal 4 CC 16, 1900 La Plata, Argentina; ∥ Universidad de Buenos Aires, Facultad de Ciencias Exactas y Naturales, Departamento de Física, Ciudad Universitaria, 1428 Buenos Aires, Argentina; ⊥ CONICET–Universidad de Buenos Aires, Instituto de Física de Buenos Aires (IFIBA), Ciudad Universitaria, 1428 Buenos Aires, Argentina; # Departamento de Química Física, Facultad de Ciencia y Tecnología, Universidad del País Vasco, Apdo. 644, E-48080 Bilbao, Spain; ∇ Departamento de Química Física, Facultad de Ciencia y Tecnología, Universidad del País Vasco, Apdo. 644, E-48080 Bilbao, Spain; ○ Department of Physics, Central Michigan University, Mount Pleasant, Michigan 48859, United States; ◆ Department of Chemistry, Rice University, Houston, Texas 77005-1892, United States; ¶ Department of Physics and Astronomy, Rice University, Houston, Texas 77005-1892, United States

## Abstract

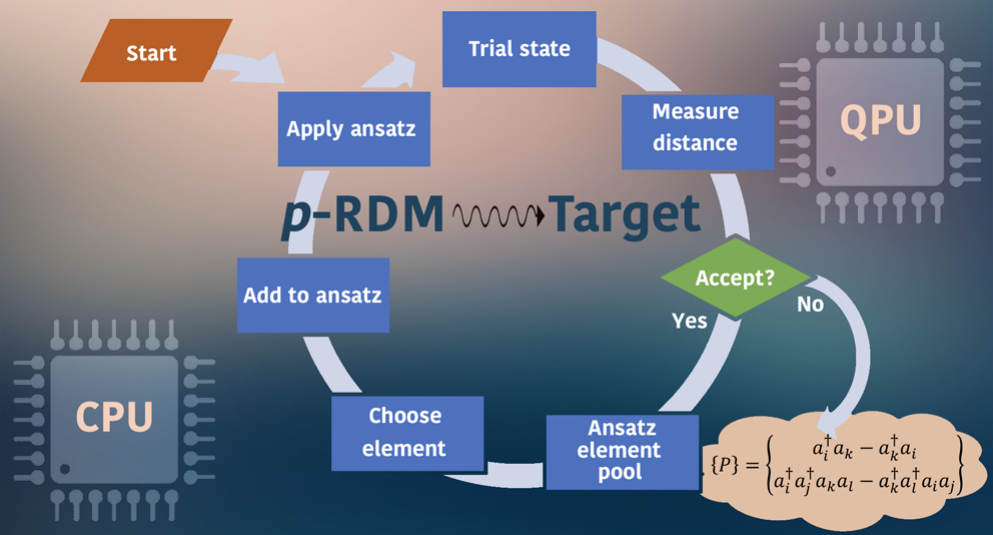

The *N*-representability problem consists in determining whether, for a given *p*-body matrix, there exists at least one *N*-body density matrix from which the *p*-body matrix can be obtained by contraction, that is, if the given matrix is a *p*-body reduced density matrix (*p*-RDM). The knowledge of all necessary and sufficient conditions for a *p*-body matrix to be *N*-representable allows the constrained minimization of a many-body Hamiltonian expectation value with respect to the *p*-body density matrix and, thus, the determination of its exact ground state. However, the number of constraints that complete the *N*-representability conditions grows exponentially with system size, and hence, the procedure quickly becomes intractable for practical applications. This work introduces a hybrid quantum-stochastic algorithm to effectively replace the *N*-representability conditions. The algorithm consists of applying to an initial *N*-body density matrix a sequence of unitary evolution operators constructed from a stochastic process that successively approaches the reduced state of the density matrix on a *p*-body subsystem, represented by a *p*-RDM, to a target *p*-body matrix, potentially a *p*-RDM. The generators of the evolution operators follow the well-known adaptive derivative-assembled pseudo-Trotter method (ADAPT), while the stochastic component is implemented by using a simulated annealing process. The resulting algorithm is independent of any underlying Hamiltonian, and it can be used to decide whether a given *p*-body matrix is *N*-representable, establishing a criterion to determine its quality and correcting it. We apply the proposed hybrid ADAPT algorithm to alleged reduced density matrices from a quantum chemistry electronic Hamiltonian, from the reduced Bardeen–Cooper–Schrieffer model with constant pairing, and from the Heisenberg XXZ spin model. In all cases, the proposed method behaves as expected for 1-RDMs and 2-RDMs, evolving the initial matrices toward different targets.

## Introduction

The *N*-representability problem has been identified as one of the most important challenges in electronic structure theory.^1^ Since only the two-body reduced density matrix (2-RDM) needs to be determined to calculate the energy of a pairwise interacting system (such as interacting electrons in atoms, molecules, or condensed matter),^2,3^ it is common to focus the *N*-representability problem on finding the necessary and sufficient constraints or conditions for the 2-RDM to ensure that it arises from the reduction of an *N*-body density matrix.^4^ These conditions are known as the *N*-representability conditions,^2−9^ and a failure to satisfy them causes a collapse of variational approaches, potentially leading to energies below their true ground state. The *N*-representability problem can be categorized as belonging to the quantum Merlin-Arthur complete class, a generalization of the (nondeterministic) polynomial-time complete class.^10^ This emphasizes its poor computational tractability for practical calculations. Recent advances in this field^9,11,12^ have enabled more accurate approximations that utilize *N*-representability conditions able to target strongly correlated systems. However, the problem’s intrinsic complexity makes this approach accessible only to a relatively small set of systems. In general, the *N*-representability problem is presented for a *p*-RDM derived from contracting (*N* – *p*) degrees of freedom of an *N*-body pure state or density matrix (single wave function) or of an *N*-body ensemble (convex linear combination) of pure states or density matrices.^2^ Although most works have been historically focused on ensemble *N*-representability, some recent papers tackle the pure *N*-representability problem.^3,11,13−21^

The adaptive derivative-assembled pseudo-Trotter (ADAPT) variational quantum eigensolver (VQE) has been introduced to enable electronic structure quantum simulations in near-term quantum hardware.^22^ Starting from an initial wave function ansatz, the algorithm evolves the ansatz by successively applying unitary transformations depending on one- and two-body operators selected from a predefined pool, while a variational parameter that minimizes the total energy is introduced at every step. One of the advantages of the ADAPT-VQE is that the number of resulting variational parameters is small, which makes its implementation in quantum computers attainable with shallow-depth quantum circuits.^22−24^ Other approaches related to the ADAPT-VQE including the contracted quantum eigensolvers (CQE)^25,26^ have been successfully applied to several problems ranging from quantum chemical simulations, strongly correlated systems, and wave function optimization.^22,27−32^

In this work, we introduce a hybrid ADAPT variational quantum algorithm (VQA) that enables an initial *p*-RDM to evolve toward a target *p*-body matrix (alleged *p*-RDM) and approach it as much as possible, using the Hilbert-Schmidt distance as a measure. Other hybrid strategies which directly determine (approximately) *N*-representable RDMs on a quantum device have also been proposed in the literature.^25,33,34^ Our proposed algorithm incorporates elements from the ADAPT methodology to properly evolve the *p*-RDM and relies on stochastic optimization to minimize the distance to avoid barren plateaus in the search process. Importantly, the proposed ADAPT-VQA is independent of any underlying Hamiltonian, can be used to determine the quality of an alleged *p*-RDM and to correct it, and is robust under statistical noise.

## Theory and Algorithm

The underlying idea of our VQA is to utilize ansatz circuits, which are characterized by a collection of vector parameters, {θ⃗}, for producing entangled trial *N*-body (pure) states or density matrices, denoted as ρ({θ⃗}). A classical stochastic global search algorithm is employed to adjust the vector parameters {θ⃗} and minimize the cost function given by the Hilbert-Schmidt distance *D* between the (physical) reduced state of ρ({θ⃗}) on a *p*-body subsystem, represented by a *p*-body reduced density matrix , and a fixed target (possibly physical) represented by ^*p*^ρ_*t*_. Provided that the ansatz is sufficiently expressive so that it contains a circuit that well-approximates the optimal solution, this distance satisfies
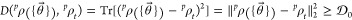
1which allows min_{θ⃗}_(*D*(^*p*^ρ({θ⃗}), ^*p*^ρ_*t*_)) to approach the minimal feasible distance . Importantly,  if and only if ^*p*^ρ_*t*_ is pure *N*-representable, and hence it can be used as a measure of the degree of *N*-representability of ^*p*^ρ_*t*_. Moreover, the evolved ρ({θ⃗}) can be used to obtain the *N*-representable (physical) reduced state ^*p*^ρ closest to ^*p*^ρ_*t*_. Since ^*p*^ρ_*t*_ is not necessarily *N*-representable, this highlights a potential use of the algorithm: to correct a non-*N*-representable matrix.

To perform minimization of the distance *D*, we took a hybrid approach. A classical stochastic optimizer iteratively constructs the ansatz generating the parametrized state, while a quantum computer calculates the Hilbert-Schmidt distance value for this parametrized state. At each iteration step *n*, the state generated by a set of parameters {θ⃗}_*n*_ can be written as
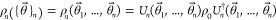
2where ρ_0_ is an initial state and *U*_*n*_ represents a unitary transformation that depends on the parameters {θ⃗}_*n*_, which results from the successive actions of single unitary transformations. Eq 2 evidences that since ρ_*n*_({θ⃗}_*n*_) is parametrized by *n* vector parameters, then the corresponding *p*-body reduced state is also parametrized by the same *n* vector parameters, ^*p*^ρ_*n*_({θ⃗}_*n*_). In our ADAPT-VQA, a stochastic optimizer, implemented on a classical computer, and a quantum algorithm, realized on a quantum computer, operate in conjunction to find a minimum-distance value of *D*:
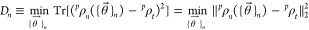
3In other words, the ADAPT-VQA gradually incorporates parametrized elements into its ansatz to form ^*p*^ρ_*n*_({θ⃗}_*n*_) such that *D*_*n*_ approaches  as *n* → ∞.

**Figure 1:**
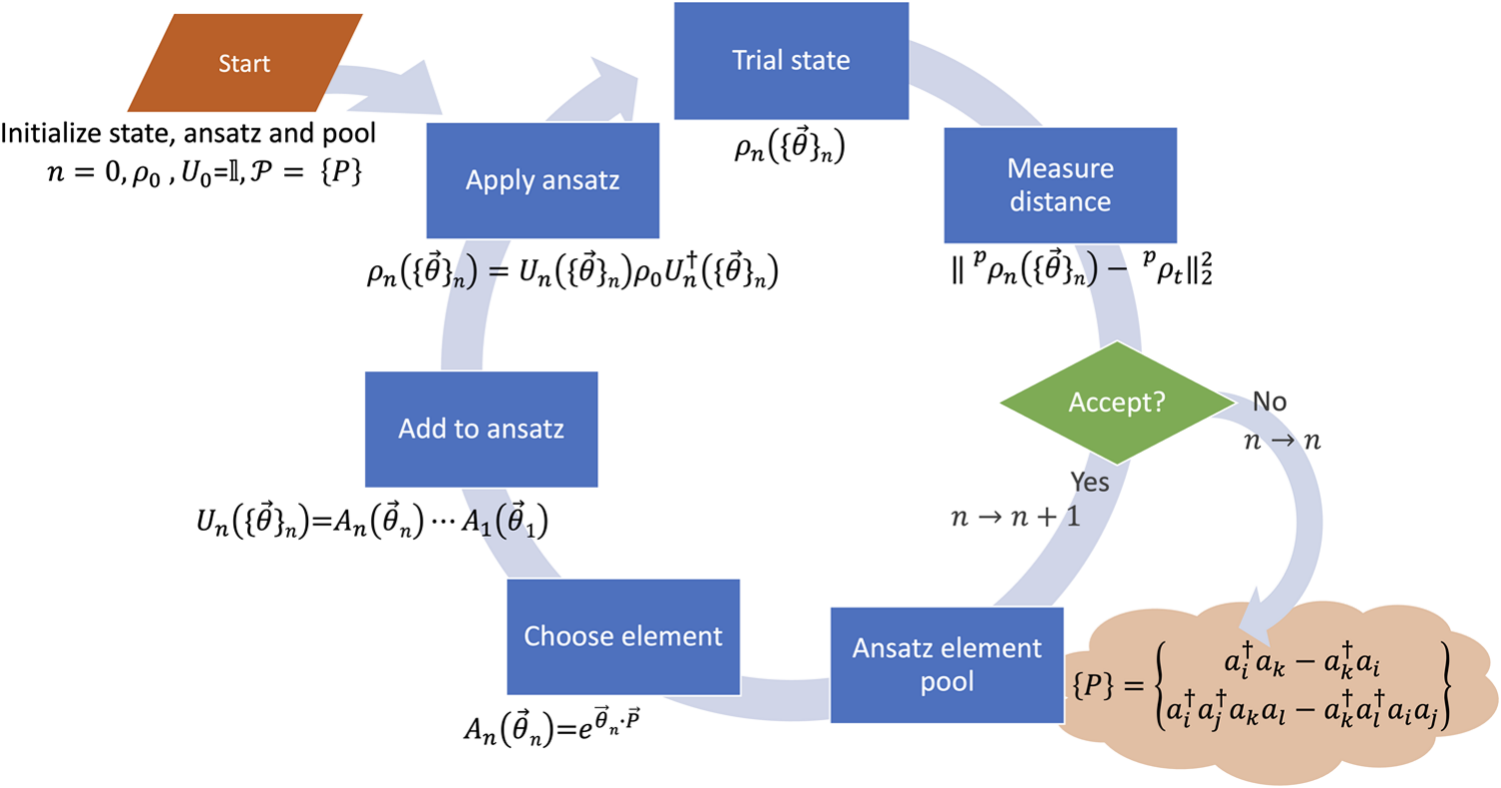
ADAPT-VQA flowchart.

The iterative ansatz is constructed by first initializing the ADAPT-VQA to a state ρ_0_, usually an independent-particle-model state, and then generating a series of trial states by adding, one at the time, elements of the form
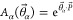
4where *P⃗* is a -dimensional vector embedding a finite pool  of antihermitian operators (see the next section), and θ⃗_α_ is a -dimensional vector parameter that has all zero elements except one randomly chosen with a random amplitude in the interval [−θ**_max_**, θ_max_], with θ_max_ decreasing or increasing as the iteration number increases depending on the local features of the cost function. Thus, the unitary ansatz evolves using the simple prescription
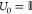
5
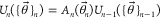
6At each iteration, the resulting unitary ansatz is accepted with a certain probability, which is initially high and gradually decreases as the number of iterations increases. This process ensures that the Hilbert-Schmidt distance *D*_*n*_ progressively decreases and potentially avoids barren plateaus found in gradient-descent methods.^35^ The distance *D*_*n*_ is evaluated in a quantum computer after adding *A*_*n*_(θ⃗_*n*_) in the *n*th step as
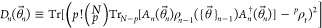
7Provided that the difference *D*_*n*_ – *D*_*n*–1_ is greater than a distance precision ϵ, the iterative algorithm proceeds. If *D*_*n*_ – *D*_*n*–1_ is less than or equal to ϵ for a certain number of consecutive steps, the algorithm terminates at a final length *n* = *L* and produces *D*_*L*_ ≡ *D*_*n*_ as the numerical estimate of . The algorithm flowchart is provided in Figure 1.

## Simulations

To explore the applicability of our ADAPT-VQA, we have chosen three representative strongly correlated systems typically used in quantum chemistry, nuclear structure, and condensed matter physics:^36−39^ the linear H_4_ molecule, the reduced Bardeen–Cooper–Schrieffer (BCS) or constant pairing model, and the Heisenberg XXZ spin model, respectively. With this choice, we also test the capability of our ADAPT-VQA with systems that are not well described by unitary coupled cluster theories.^40,41^

In this work, we focus on a Fermionic ADAPT-VQA where the pool of excitation operators can be reduced to^42−45^
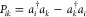
8and
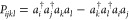
9Here, *a*_*i*_^†^ and *a*_*i*_ are Fermion creation and annihilation operators acting on an orthonormal finite single-particle basis {*i*, *j*, *k*, *l*, ···}, represented in terms of Pauli operators using the Jordan–Wigner transformation as is paramount to simulate Fermionic systems on quantum devices.^46^

### Computational Details

All of the computations in this work were carried out simulating an ideal, noiseless quantum device using an in-house code that interfaces PySCF(47,48) and the OpenFermion module of PySCF for integral computations and the OpenFermion code^49^ for the Jordan–Wigner mappings. The pool of operators consists of spin-adapted generalized singles and doubles, with double excitation operators decomposed into singlets and triplets.^22,50^ This choice, which is due to the nature of the treated systems, significantly reduces the number of operators in the pool for hard-core bosons and spin models based on the *SU*(2) pairing algebra. All calculations involving electrons were performed using the STO-3G basis set.^51^

The stochastic optimization uses the simulated annealing (SA) algorithm introduced by Kirkpatrick et al.^52^ The SA algorithm consists of performing a sequence of stochastic configuration sampling of the search space using an acceptance criterion of the new configuration based on the Boltzmann probability distribution at temperature *T* while progressively reducing *T*. A system in thermal equilibrium at temperature *T* can be found in a state with a cost function (the Hilbert-Schmidt distance in our case) *D* with a probability proportional to exp(−*D*/*T*). At low temperatures, there is a small probability that the system will be in a high-cost-function state. This plays a crucial role in the procedure because a possible increase in the cost function allows the system to escape from the local minima and potentially find the global minimum. According to our experience, a good choice for *T* is in the range [0, 0.1] (atomic units will be used throughout) depending on the system and target *p*-body matrix, allowing to avoid possible local minima and, at the same time, keeping the number of iterations manageable. The decreasing rate of *T* for each step *i* is chosen so that *T*^(*i*+1)^ = *δTT*^(*i*)^ with *δT* = 0.995, while θ_max_^(*i*+1)^ = δθ_max_ θ_max_^(*i*)^, with an initial value θ_max_ = 0.5 and δθ_max_ = 0.999 when the new configuration is not accepted or δθ_max_ = 1.0025 otherwise. This strategy effectively decreases θ_max_ slowly, while still allowing the system to escape local minima.

The variational two-body-reduced-density-matrix (v2RDM) methods relying on semidefinite programming^2,3,53−59^ within a SU(2) pairing algebra, or doubly occupied configuration interaction (DOCI) framework,^36,37,54,60−64^ with different *N*-representability conditions utilized to obtain (non-*N*-representable) approximated two-body reduced density matrices of the ground state of the reduced BCS and Heisenberg XXZ spin models. The resulting matrices were then used as targets for the ADAPT-VQA to analyze the numerical effectiveness of the method by comparing the v2RDM solutions. The v2RDM method provides a lower-bound energy to the exact ground state, and it has been utilized to validate different sets of *N*-representability conditions.^54^ The constraints typically utilized in v2RDM methods are not restrictive enough to ensure the exactness of the resulting two-body matrices, hereafter denoted as ^2^ρ_v2RDM_. In our case, we obtain the ^2^ρ_v2RDM_ applying two-positivity and partial three-positivity T1 and T2′ conditions^55,65−67^ reformulated using the SU(2) pairing algebra,^36,37,60−64^ which are two of the most restrictive sets of constraints currently available for practical calculations, with the latter leading to a more stringent combined set and therefore significantly improving the accuracy of the two-positivity conditions.^9^ These two sets of constraints, hereafter denoted as 2-POS and (2,3)-POS, are complemented with hermiticity and normalization conditions on ^2^ρ_v2RDM_, and with the corresponding contraction and consistency relations for each set of conditions.^36,37,62,63^

### Linear H_4_ Molecule

Within the Born–Oppenheimer approximation, the nonrelativistic Hamiltonian of an *N*-electron molecular system can be written within the second quantization formalism as^68^
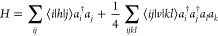
10where ⟨*i*|*h*|*j*⟩ and ⟨*ij*|*v*|*kl*⟩ are the standard one- and two-electron antisymmetrized integrals, respectively, and *a*_*i*_^†^ and *a*_*j*_ are the Fermion creation and annihilation operators acting on an orthonormal 2K-dimensional single-particle spin–orbital basis built from K spatial orbitals. For the case of the linear H_4_ molecule at 0.75 Å interatomic separation, the exact (within the basis set) singlet ground- and first-excited full configuration interaction solutions are used to obtain the corresponding exact one-body reduced density matrices, denoted as ^1^ρ_exact_, and the exact ground-state two-body reduced density matrix, denoted as ^2^ρ_exact_, respectively. The resulting ground state resembles a closed-shell singlet with a single leading determinant coefficient of ∼0.98, while the first excited state is of multireference character, closely resembling a spin-adapted singlet with two dominating determinants with the largest coefficient of ∼0.70. The eigenvalues of the exact ground state 1-RDM lie in the interval [0.0048,0.9936], while those for the excited state are in the interval [0.0015, 0.9985]. On the other hand, the eigenvalues of the exact ground-state 2-RDM were found in the interval [0.0000,2.0148].

**Figure 2:**
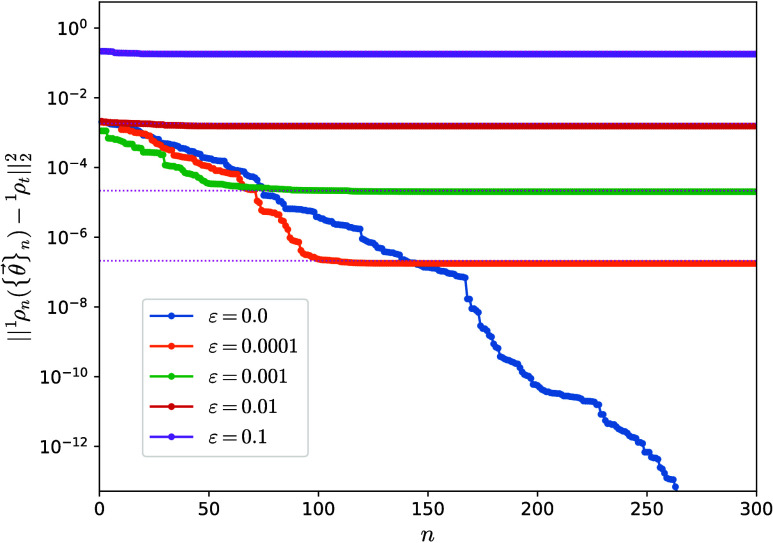
Distance between the one-body reduced density matrix ^1^ρ({θ⃗}) and a fixed noisy target ^1^ρ_*t*_ as a function of iteration number *n* for the linear H_4_ molecule. The target is constructed by adding random noise of strength ε to the exact ground-state ^1^ρ_exact_. Distances between the exact (unperturbed) ground-state one-body reduced density matrix and the fixed targets ^1^ρ_*t*_ are shown as upper-bound references with pink dotted lines. The initial ρ_0_ is constructed from the Hartree–Fock ground state.

Our first test was to monitor the evolution of the distance *D* between the (physical) reduced state of ρ({θ⃗}) on a one-body subsystem, represented by a one-body reduced density matrix ^1^ρ({θ⃗})≡ *N*Tr_*N*–1_[ρ({θ⃗})] and a fixed target one, represented by ^1^ρ_*t*_, as a function of iteration number *n*. We build a noisy target one-body matrix from ^1^ρ_exact_ as ^1^ρ_*t*_ = ^1^ρ_exact_ + ε *R*, with *R* a matrix of random numbers taken from a uniform probability distribution in [−1, 1] and ε the strength of perturbation in the interval [0, 0.1], which must be compared with the extreme eigenvalues of the (unperturbed) exact 1-RDM. The ADAPT-VQA is expected to evolve the reduced state of ρ_0_, initialized as the Hartree–Fock ground state, on a one-body subsystem toward the noisy ^1^ρ_*t*_. Since the noise in the target breaks the *N*-representability of the exact 1-RDM, the algorithm should approach ^1^ρ_*n*_ only to the target up to a certain limit, as seen in Figure 2. Larger noise strengths ε lead to convergence of the algorithm to larger *D*, while a noiseless target allows ^1^ρ_*n*_ to approach ^1^ρ_*t*_ to 10^–12^ in ∼250 iterations.

**Figure 3:**
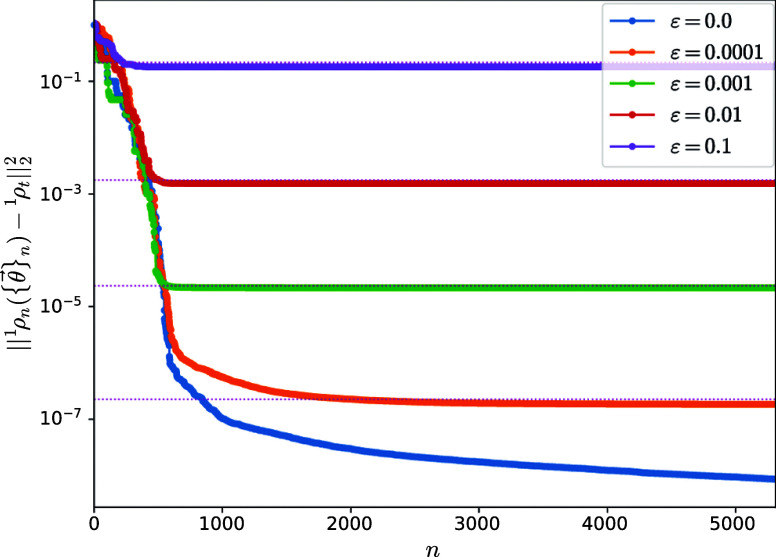
Distance between the one-body reduced density matrix ^1^ρ({θ⃗}) and a fixed noisy target ^1^ρ_*t*_ as a function of iteration number *n* for the linear H_4_ molecule. The target is constructed by adding random noise of strength ε to the exact first excited state ^1^ρ_exact_. Distances between the exact (unperturbed) first-excited-state one-body reduced density matrix and the fixed targets ^1^ρ_*t*_ are shown as upper-bound references with pink dotted lines. The initial ρ_0_ is constructed from the Hartree–Fock ground state.

As a second test, we repeated the previous numerical experiment but using the one-body reduced density matrix corresponding to the exact first-excited state, also denoted as ^1^ρ_exact_, while keeping ρ_0_ as the Hartree–Fock ground state. Perturbation strength ϵ was also considered the interval [0, 0.1], which must be compared against the extreme eigenvalues of the (unperturbed) exact 1-RDM. Here, one expects a slower convergence, as shown in Figure 3, but nonetheless, the ADAPT-VQA performs as expected. Approximately 6000 simulated annealing iterations are needed to reach a distance of 10^–8^ from the noiseless target.

Importantly, when the ADAPT-VQA is initialized with a correlated state, the algorithm converges to the same solution obtained with the Hartree–Fock state for both cases, the ground and excited target matrices (see the Supporting Information for details).

**Figure 4:**
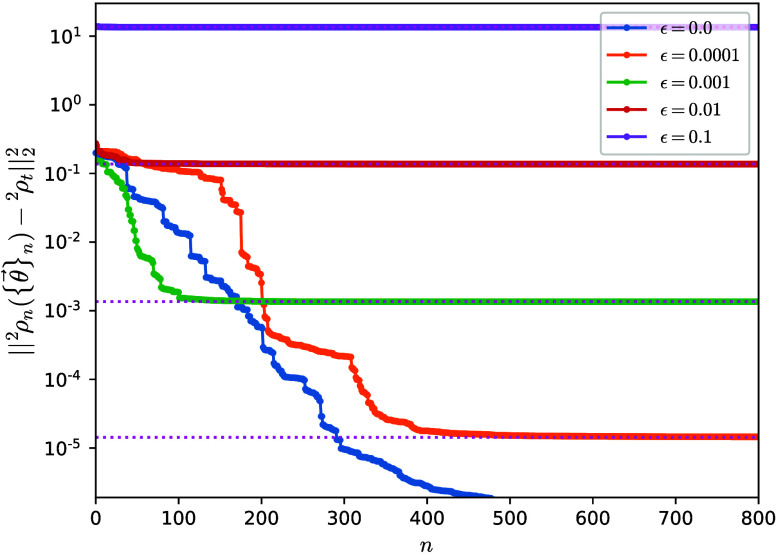
Distance between the two-body reduced density matrix ^2^ρ({θ⃗}) and a fixed noisy target ^2^ρ_*t*_ as a function of iteration number *n* for the linear H_4_ molecule. The target is constructed by adding random noise of strength ε to the exact ground-state ^2^ρ_exact_. Distances between the exact (unperturbed) ground-state two-body reduced density matrix and the fixed targets ^2^ρ_*t*_ are shown as upper-bound references with pink dotted lines. The initial ρ_0_ is constructed from the Hartree–Fock ground state.

Next, we examine the behavior of our ADAPT-VQA for two-body reduced density matrices. To this end, we analyze the convergence of ^2^ρ_*n*_ toward the ^2^ρ_exact_ with noise added as in the two cases before, which must be compared against the extreme eigenvalues of the (unperturbed) exact 2-RDM. Again, ρ_0_ is initialized as the Hartree–Fock ground state. The stochastic process allows ^2^ρ_*n*_ to approach the target 2-RDM progressively up to a certain limit determined by the level of noise in the target, as shown in Figure 4. The noiseless case reaches a value of *D* ∼10^–5^ in ∼300 iterations. Here it should be noted that the dimension of the 2-RDM problem is larger than that corresponding to the 1-RDM, and thus a larger number of simulated annealing steps are expected to reach similar convergence. The three examples reported for the H_4_ molecule show that the algorithm works as expected for electronic systems.

### Reduced BCS Model

The reduced BCS or constant pairing model is employed in nuclear^69^ and condensed matter physics^70,71^ to model the superfluid and superconducting properties of finite and extended systems, respectively. In this work, we use the reduced BCS Hamiltonian in the form^36,72^
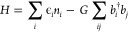
11where *G* is the strength of the infinite-range pairing interaction, which may be repulsive (*G* < 0) or attractive (*G* > 0), and ϵ_*i*_ are single-particle energies. The number operator is , and the pair-creation (annihilation) operators are *b*_*i*_^†^ = (*b*_*i*_)^†^ = *a*_*i*_^†^*a*_*i̅*_^†^. We will only consider half-filled states with the number of pairs M = *K*/2 (where *K* is the total number of single-particle levels) and equally spaced single-particle energies ε_*i*_ = *i*/*K*, with *i* = 1, 2, ···, *K*. The (*i*, *i̅*) pair defines the pairing scheme, which involves two particles with opposite conjugate quantum numbers (i.e., spin or momentum) in doubly degenerate single-particle levels. The Hamiltonian (11) is based on the *SU*(2) pairing algebra with generators *b*_*i*_^†^ and (2*n*_*i*_ – 1)/2, and hard-core boson relations^73,74^
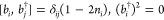
12

**Figure 5:**
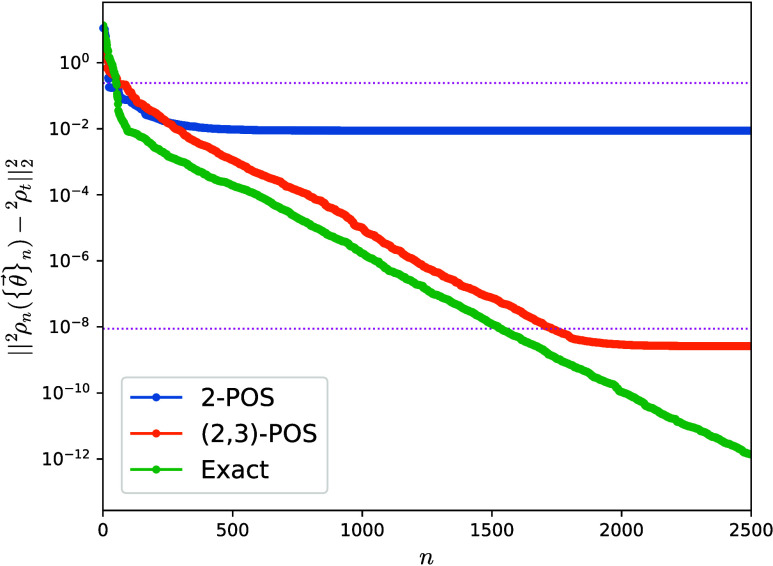
Distance between the two-body reduced density matrix ^2^ρ({θ⃗}) in the reduced BCS model (see text) and three different targets obtained from the v2RDM methods with 2-POS and (2,3)-POS conditions and the exact ground-state solution. Distances between the exact ground-state two-body reduced density matrix and the fixed targets ^2^ρ_*t*_ are shown as upper-bound references with pink dotted lines. The initial ρ_0_ is constructed from the mean-field *G* = 0 pairing model ground state.

This model can be solved exactly,^72^ predicting a gapless phase (metallic) and a finite gap phase (superconducting). The critical value of the strength parameter *G* that separates these phases, *G*_c_, is obtained from the gap equation in the zero-gap limit and the chemical potential μ = (ε_*M*_ + ε_*M*+1_)/2,
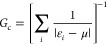
13For the reduced BCS model, we first explore the convergence of ^2^ρ_*n*_ toward a target two-body matrix calculated using three different approaches: The v2RDM methods enforce 2-POS and (2,3)-POS conditions, and the 2-RDM corresponding to the exact ground-state. These simulations employ an interaction strength *G* = 1 for a system of *K* = 4 single-particle levels at half-filling, and the initial ρ_0_ is initialized from a simple independent-particle-model solution, the noninteracting *G* = 0 pairing model ground state. In this case, the BCS model with a critical value, *G*_c_ = 0.1875, is a sensible example to assess the extent of pairing correlations. Hence, we have considered pairing strengths around this value. As in the previous cases, our stochastic ADAPT-VQA evolves ^2^ρ_*n*_ toward the target matrix, Figure 5. In the case of the exact ground-state target, the distance reduces to 10^–12^ in about 2500 iterations, while with targets obtained from the v2RDM methods, the distance plateaus to small, yet numerically significant values: about 10^–8^ for the (2,3)-POS and 10^–2^ for the 2-POS. This illustrates the potential of our approach as a tool to quantify the quality of the approximated reduced density matrices provided by different approaches.

**Figure 6:**
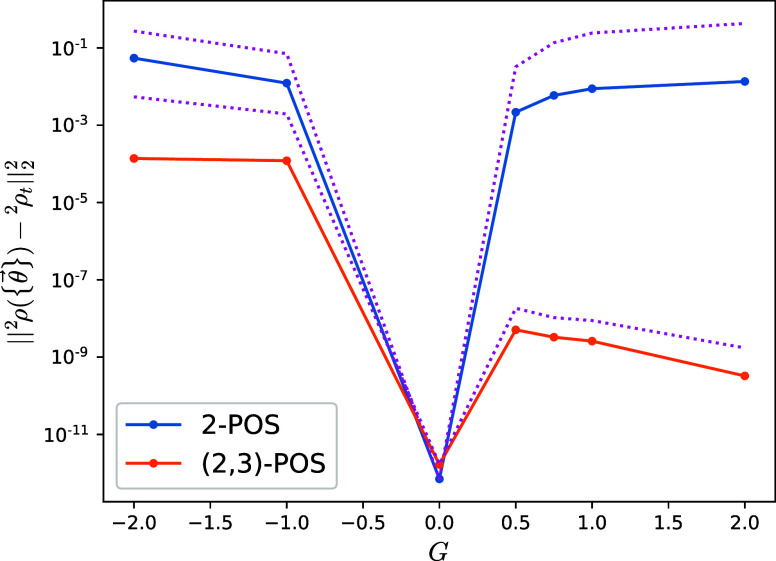
Distance between the fully evolved two-body reduced density matrix obtained with the ADAPT-VQA and a fixed target calculated from the 2-POS and (2,3)-POS v2RDM methods for the reduced BCS model. Distances between the exact ground-state two-body reduced density matrix and the fixed targets ^2^ρ_*t*_ are shown as upper-bound references with pink dotted lines. The initial ρ_0_ is constructed from the mean-field *G* = 0 pairing model ground state.

As a second test for the reduced BCS model, we now analyze the converged distance that ADAPT-VQA gives for interaction strengths *G* in the range of [−2,2]. Here, we include *G* = 0 (noninteracting) and *G* = 0.5, 0.75, ± 1, ± 2 and plot in Figure 6 the final converged distance. The v2RDM 2-RDM with 2-POS and (2,3)-POS conditions are targets, starting from the noninteracting (*G* = 0) state for the different *G* values. The ADAPT-VQA can evolve the noninteracting 2-RDM toward the approximate variational solutions for different interaction strengths. The 2-POS target shows a rather symmetric behavior around *G* = 0, with a large converged distance in the vicinity of 10^–1^ for *G* ≠ 0. On the other hand, the (2,3)-POS target shows an asymmetric behavior about *G* = 0, with larger distances of ∼10^–4^ for *G* < 0 and significantly reduced converged distances for *G* > 0, ∼10^–9^. Figure 6 shows that both 2-POS and (2,3)-POS target 2-RDMs corresponding to *G* = 0 are numerically *N*-representable, in agreement with the fact that for *G* = 0 the set of 2-POS (and consequently (2,3)-POS) *N*-representability conditions together with the hermiticity, contraction, and consistency conditions become necessary and sufficient, while including correlation (*G* ≠ 0) brings the 2-POS and (2,3)-POS targets farther from an *N*-representable 2-RDM. This highlights the potential use of the ADAPT-VQA to establish a numerical measure of *N*-representability.

### Heisenberg XXZ Model

**Figure 7:**
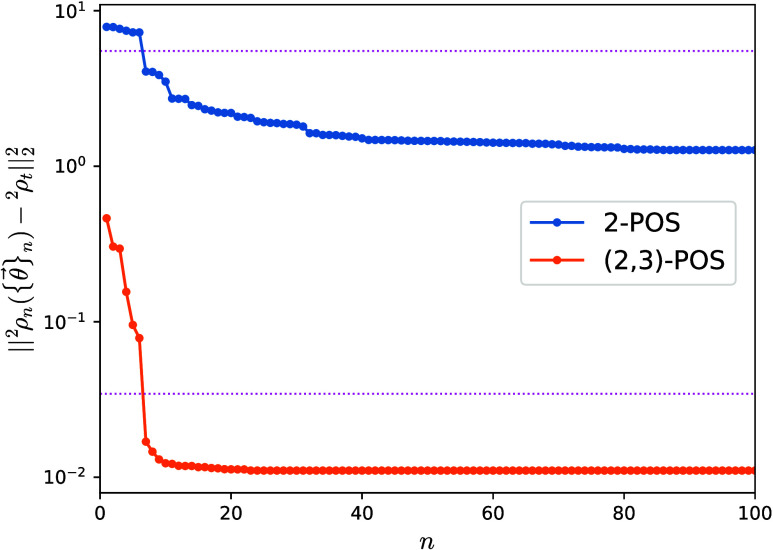
Distance between the two-body reduced density matrix ^2^ρ({θ⃗}) in the XXZ spin model with anisotropy Δ = 2 (see text) and two target matrices obtained from the v2RDM methods with 2-POS and (2,3)-POS conditions. Distances between the exact ground-state two-body reduced density matrix and the fixed targets ^2^ρ_*t*_ are shown as upper-bound references with pink dotted lines. The initial ρ_0_ is chosen as the exact ground state of the Δ = ∞ XXZ model.

The Heisenberg Hamiltonian can be used to model the low-excitation physics of a number of problems, such as quantum magnetism and Mott phase transitions.^75^ The Hamiltonian with anisotropy of XXZ type for a chain of *K* spin sites with open boundary conditions with first-neighbor interactions can be cast as
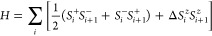
14where *S*_*i*_^±^ and *S*_*i*_^*z*^ are the Fermionic spin-ladder and spin projection operators acting on site *i*, and the parameter Δ sets the anisotropy of the model. We consider a finite chain of 4 sites at *S*_tot_^*z*^ = 0 with open boundary conditions. This model has a rich phase diagram as a function of Δ. For −1 < Δ < 1, the system is a critical antiferromagnet with gapless excitations. For |Δ| > 1, the system shows a finite energy gap, and it is ferromagnetic for Δ < −1 and antiferromagnetic for Δ > 1. Although the Hamiltonian in eq 14 can be straightforwardly implemented in a quantum computer, we opt here to map it to a hard-core bosons representation as introduced by Holstein and Primakoff.^76^ This choice allows us to directly assess the quality of variational 2-RDMs obtained using an *SU*(2) pairing algebra formalism.^37,77^ Using this representation, the spin operators are
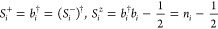
15and the total number of hard-core bosons relates to the spin *z* projection as ∑_*i*_*n*_*i*_ = M = *S*_tot_^*z*^ + *K*/2, making half-filling (*M*/*K* = 1/2) correspond to *S*_tot_^*z*^ = 0. Using this transformation, the Hamiltonian reads
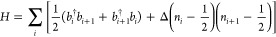
16Similar to the case of the reduced BCS model, in this case, we first analyze the convergence of a 2-RDM evolved with our ADAPT-VQA toward fixed targets. The targets are chosen as the 2-POS and (2,3)-POS v2RDM solutions as before, while the initial state ρ_0_ is initialized as the Δ = ∞ exact ground state. The results for Δ = 2 are summarized in Figure 7. In this case, convergence is achieved faster than in all of the previous numerical tests. However, it becomes evident from Figure 7 that the 2-POS and (2,3)-POS v2RDM reduced density matrices are somewhat poor approximations in terms of the quality of the matrices, giving converged distances of ∼1 and ∼10^–2^, respectively. Other authors have observed this behavior for the XXZ model using semidefinite relaxation variational RDM techniques.^37,77−79^

**Figure 8:**
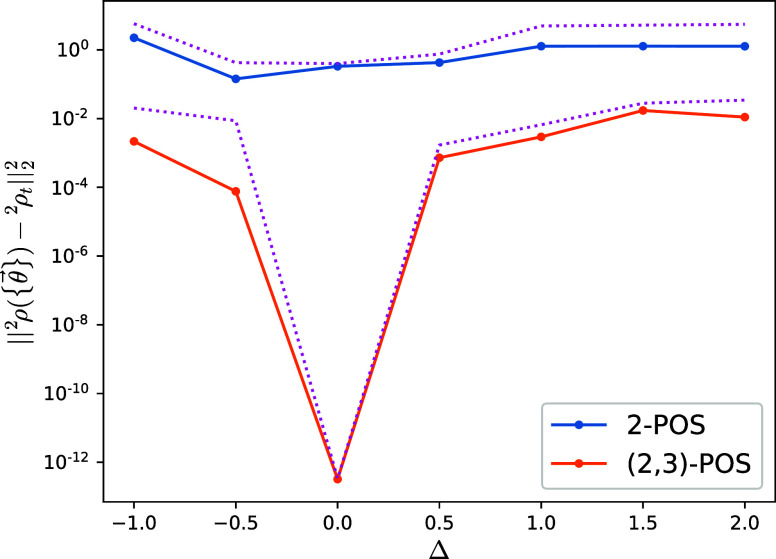
Distance between the fully evolved two-body reduced density matrices obtained with the ADAPT-VQA and fixed targets calculated from the v2RDM methods using 2-POS and (2,3)-POS conditions of the XXZ spin model as a function of the anisotropy Δ. The targets are the two-body reduced density matrices computed variationally with two-positivity (2-POS) and partial three-positivity ((2,3)-POS) conditions. Distances between the exact ground-state two-body reduced density matrix and the fixed targets ^2^ρ_*t*_ are shown as upper-bound references with pink dotted lines.

As a last test, we compare the fully converged two-body reduced density matrix distance given by our ADAPT-VQA for different anisotropy strengths Δ. We utilize, as before, the two target variational approximate two-body reduced density matrices obtained from the v2RDM methods with 2-POS and (2,3)-POS conditions. Figure 8 shows the results for −1 < Δ < 2. For the case of Δ = 0, which corresponds to the isotropic model, ADAPT-VQA can evolve the initial 2-RDM to a numerically negligible distance from the (2,3)-POS v2RDM matrix. This is not the case for other anisotropy values and for the 2-POS v2RDM matrix. There, the ADAPT-VQA evolves the initial 2-RDM to distances of ∼1 and 10^–2^ to 10^–4^ for the 2-POS and (2,3)-POS cases, respectively.

## Summary and Outlook

We have introduced a hybrid ADAPT-VQA that evolves an initial *N*-body density matrix to successively approach the reduced state of the density matrix on a *p*-body subsystem, represented by a *p*-RDM, to a target *p*-body matrix, alleged *p*-RDM. The algorithm makes use of the ADAPT methodology to progressively construct a quantum circuit, represented by a set of vector parameters and operators to generate an *N*-body density matrix. The optimization relies on a stochastic simulated annealing procedure, thus avoiding potential barren plateaus that are prone to gradient-descent methods. Importantly, the proposed ADAPT-VQA is independent of any underlying Hamiltonian and can be used to determine the quality and correct an alleged *p*-body reduced density matrix. It is robust under statistical noise.

We have challenged the robustness of the proposed ADAPT-VQA with 1- and 2-RDMs using the linear H_4_ quantum chemistry electronic Hamiltonian, the reduced BCS model with constant pairing, and the Heisenberg XXZ spin model. Using these proof-of-concept cases, we illustrate the applicability of the algorithm by considering starting points and different targets. We found that the ADAPT-VQA behaves as expected for pure 1- and 2-RDMs, evolving the initial matrices toward the target. The extension of the algorithm to evolve initial ensemble states and their corresponding *p*-RDMs, and hence to tackle the ensemble *N*-representability problem, will be reported elsewhere. Overall, the proposed ADAPT-VQA provides a valuable addition to the arsenal of electronic structure quantum algorithms.

## Supporting Information Available

Comparison of the convergence behavior of the ADAPT-VQA for different initial states (PDF)
