# Supplementary material for: Determining the N-Representability of a Reduced Density Matrix via Unitary Evolution and Stochastic Sampling
Source: J Chem Theory Comput. 2024 Nov 14;20(22):9968–76. doi: 10.1021/acs.jctc.4c01166 (PMC11603619; doi:10.1021/acs.jctc.4c01166)
Supplement: Supplementary file 1 — ct4c01166_si_001.pdf [file ct4c01166_si_001.pdf]

# Supporting Information: Determining the $N$-representability of a reduced density matrix via unitary evolution and stochastic sampling


Gustavo E. Massaccesi,[†,‡] Ofelia B. Oña,[¶] Pablo Capuzzi,[§,‖] Juan I. Melo,[§,‖] Luis Lain,[⊥] Alicia Torre,[#] Juan E. Peralta,[*,@] Diego R. Alcoba,[*,§,‖] and Gustavo E. Scuseria[*,△,∇]

†*Departamento de Ciencias Exactas, Ciclo Básico Común, Universidad de Buenos Aires, Ciudad Universitaria, 1428 Buenos Aires, Argentina*

‡*Instituto de Investigaciones Matemáticas "Luis A. Santaló" (IMAS), Consejo Nacional de Investigaciones Científicas y Técnicas, Universidad de Buenos Aires. Ciudad Universitaria, 1428 Buenos Aires, Argentina*

¶*Instituto de Investigaciones Fisicoquímicas Teóricas y Aplicadas, Universidad Nacional de La Plata, Consejo Nacional de Investigaciones Científicas y Técnicas. Diag. 113 y 64 (S/N), Sucursal 4, CC 16, 1900 La Plata, Argentina*

§*Universidad de Buenos Aires, Facultad de Ciencias Exactas y Naturales, Departamento de Física. Ciudad Universitaria, 1428 Buenos Aires, Argentina*

‖*CONICET - Universidad de Buenos Aires, Instituto de Física de Buenos Aires (IFIBA). Ciudad Universitaria, 1428 Buenos Aires, Argentina*

⊥*Departamento de Química Física, Facultad de Ciencia y Tecnología, Universidad del País Vasco. Apdo. 644, E-48080 Bilbao, Spain*

#*Departamento de Química Física, Facultad de Ciencia y Tecnología, Universidad del País Vasco, Apdo. 644, E-48080 Bilbao, Spain*

@*Department of Physics, Central Michigan University, Mount Pleasant, MI, 48859, USA*

△*Department of Chemistry, Rice University, Houston, TX 77005-1892*

∇*Department of Physics and Astronomy, Rice University, Houston, TX 77005-1892*

# Contents

Figure S1 .......... S-2

Figure S2 .......... S-3

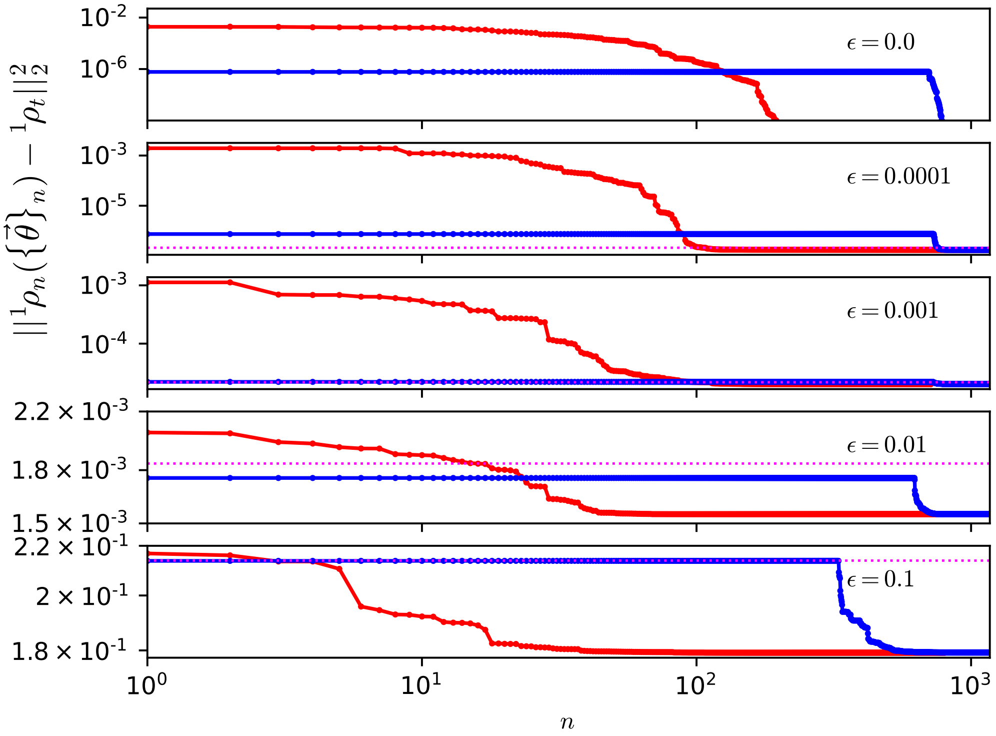


Figure S1: Distance between the 1-body reduced density matrix ${}^1\rho(\{\vec{\theta}\})$ and a fixed noisy target ${}^1\rho_t$ as a function of iteration number $n$ for the linear $H_4$ molecule. The target is constructed by adding random noise of strength $\varepsilon$ to the exact ground-state ${}^1\rho_{\text{exact}}$. Distances between the exact (unperturbed) ground-state 1-body reduced density matrix and the fixed targets ${}^1\rho_t$ are shown as upper-bound references with pink dotted lines. The initial $\rho_0$ is constructed from the Hartree-Fock (in red) and single and double excitation configuration interaction (in blue) ground states.

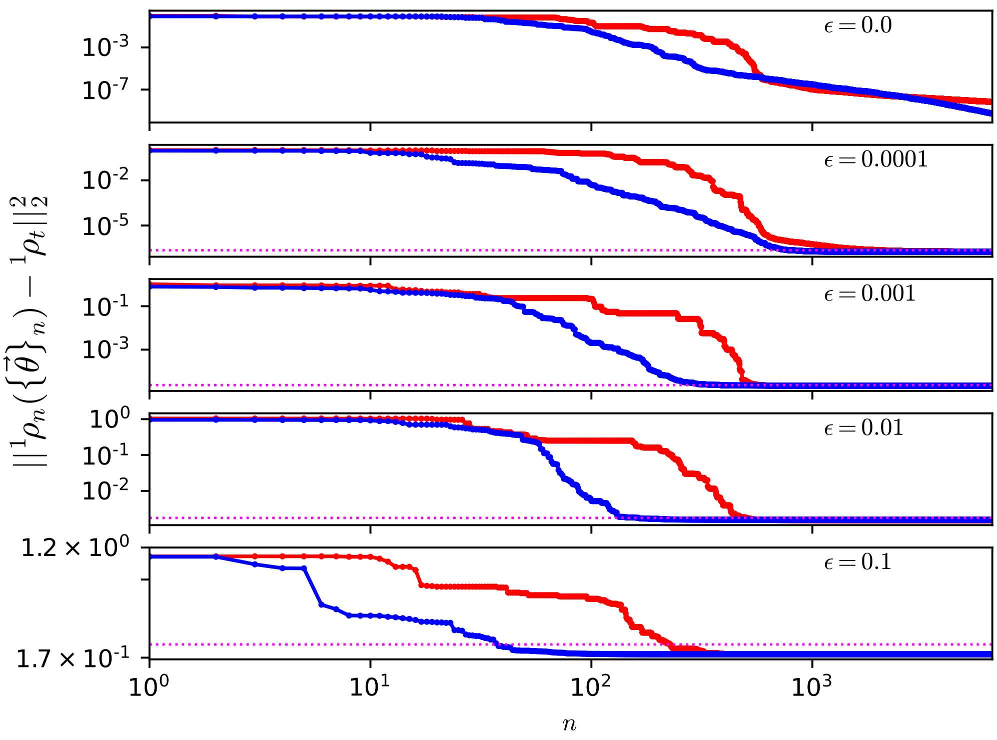


Figure S2: Distance between the 1-body reduced density matrix ${}^1\rho(\{\vec{\theta}\})$ and a fixed noisy target ${}^1\rho_t$ as a function of iteration number $n$ for the linear $H_4$ molecule. The target is constructed by adding random noise of strength $\varepsilon$ to the exact first excited state ${}^1\rho_{\mathrm{exact}}$. Distances between the exact (unperturbed) first-excited-state 1-body reduced density matrix and the fixed targets ${}^1\rho_t$ are shown as upper-bound references with pink dotted lines. The initial $\rho_0$ is constructed from the Hartree-Fock (in red) and single and double excitation configuration interaction (in blue) ground states.
